# Critical connectivity for emergence of collective oscillations in strongly diluted neural networks

**DOI:** 10.1186/1471-2202-14-S1-P394

**Published:** 2013-07-08

**Authors:** Stefano Luccioli, Simona Olmi, Antonio Politi, Alessandro Torcini

**Affiliations:** 1 CNR - Consiglio Nazionale delle Ricerche - Istituto dei Sistemi Complessi, via Madonna del Piano 10, I-50019 Sesto Fiorentino, Italy; 2 INFN Sez. Firenze, via Sansone, 1 - I-50019 Sesto Fiorentino, Italy; 3 Centro Interdipartimentale per lo Studio delle Dinamiche Complesse, via Sansone, 1 - I-50019 Sesto Fiorentino, Italy; 4 Institute for Complex Systems and Mathematical Biology and SUPA, University of Aberdeen, Aberdeen AB24 3UE, UK

## 

Among the most relevant dynamical phenomena observed in brain circuits is the rhythmic collective behavior of neuronal populations [1]. In this work [2] we studied the dynamics of random neural networks models focusing on the role played by the (in-degree) connectivity K (i.e., the number of incoming connections per node) on the onset of collective oscillations. In modeling neural networks two classes of systems are generally considered [3]: massive networks, where K is proportional to the network size N; *sparse *(or *strongly diluted*) networks, where K<< N, and specifically K is independent on N as N → ∞.

While it is not surprising to observe the onset of a collective motion in massive networks, it is less obvious to predict whether and when this can happen in sparse ones. Here, we showed that a *finite critical connectivity *Kc is able to sustain the emergence of collective oscillations and that this is a general and robust property of *sparse *networks. Since Kc turns out to be surprisingly of the order of a few tens in all models we have investigated, macroscopic motion appears to be rather ubiquitous and relevant in the context of neural dynamics. The existence of a critical connectivity separating asynchronous from coherent activity is similar to what experimentally observed in neuronal cultures [4].

Moreover, we showed that the microscopic evolution of sparse networks is extensive (i.e. the number of active degrees of freedom is proportional to the number of network elements) according to what observed for the Θ-neuron model in Ref. [5]. This property is highly nontrivial, as the dynamics of a sparse network is intrinsically non additive [6] (it cannot be approximated with the juxtaposition of almost indepedent sub-structures).

We found all the above striking results to hold for networks of pulse-coupled leaky-integrate-and-fire neurons, among the most popular and yet simple models used in computational neuroscience, and more generally also for other kinds of networks (chaotic maps and Stuart-Landau oscillators).
